# The Role of miRNAs in Common Inflammatory Arthropathies: Osteoarthritis and Gouty Arthritis

**DOI:** 10.3390/biom6040044

**Published:** 2016-11-11

**Authors:** Panagiota Papanagnou, Theodora Stivarou, Maria Tsironi

**Affiliations:** 1 Department of Nursing, Faculty of Human Movement and Quality of Life Sciences, University of Peloponnese, Orthias Artemidos and Plateon St, GR-23100 Sparti, Greece; theodorast@gmail.com (T.S.); tsironi@uop.gr (M.T.); 2 Immunology Laboratory, Immunology Department, Hellenic Pasteur Institute, P.O Box 115 21, Athens, Greece

**Keywords:** arthropathy, post-transcriptional regulator, metaflammation, overweight, cartilage erosion, hyperuricemia, monosodium urate crystals

## Abstract

MicroRNAs (miRNAs) are small, non-coding RNA species that are highly evolutionarily conserved, from higher invertebrates to man. Up to 1000 miRNAs have been identified in human cells thus far, where they are key regulators of the expression of numerous targets at the post-transcriptional level. They are implicated in various processes, including cell differentiation, metabolism, and inflammation. An expanding list of miRNAs is known to be involved in the pathogenesis of common, non-autoimmune inflammatory diseases. Interestingly, osteoarthritis (OA) is now being conceptualized as a metabolic disease, as there is a correlation among hyperuricemia and metabolic syndrome (MetS). Experimental evidence suggests that metabolic deregulation is a commonality between these different pathological entities, and that miRNAs are key players in the modulation of metabolic routes. In light of these findings, this review discusses the role of miRNAs in OA and gouty arthritis, as well as the possible therapeutic targetability of miRNAs in these diseases.

## 1. Introduction

Functionally, critical responses, such as inflammatory ones, are subjected to strict control, not only through the “supervising” role of modulatory proteins, but also by distinct RNA species, which, although they do not code for any protein, are key regulators for many functional systems, including the immune system. These non-coding, regulatory RNA species are the so-called microRNAs (hereafter referred to as “miRNAs”), which function as post-transcriptional regulators of gene expression. miRNAs can variably regulate the expression of numerous, even hundreds of different transcripts. In turn, the expression of a certain transcript may be controlled by multiple miRNAs [1,2,3,4,5].

miRNAs are short, single-stranded molecules displaying remarkable evolutionary conservation, from fruit fly (*Drosophila melanogaster*) to man [6]. Cells of human origin express up to 1000 miRNAs, with their expression patterns being cell/tissue-specific or not [7,8,9,10]. Genes coding for miRNAs may be found as independent genes, or they may map to intronic regions of other genes, and they are transcribed by RNA polymerases II and III. Adding another level of complexity, miRNA transcription units may be either mono- or poly-cistronic [11].

Biogenetically, miRNAs are first produced in the nucleus, in the form of primary miRNAs (pri-miRNAs), which undergo two consecutive steps of ribonucleolytic processing by two different RNases III in a cellular compartment-specific manner, i.e., by Drosha, within the nucleus, and then by Dicer, in the cytosol [12]. The first Drosha-mediated step results in the production of a hairpin-like precursor miRNA (pre-miRNA) that is exported to the cytosol in an exportin-5-dependent fashion. In the cytosol, Dicer, along with other factors, further processes pre-miRNA into a shorter (up to 24 nt) double-stranded molecule, of which the “guide strand” is eventually incorporated into a multicomponent ribonucleoprotein complex termed the RNA-induced silencing complex (RISC) and the other strand (symbolized as miRNA*) is degraded [11,13,14,15,16]. As part of the RISC, the “seed” region of a miRNA imperfectly forms base pairs with the 3′ untranslated region (UTR) of mRNA target(s) to shut off expression in various ways, including inhibition of translation and mRNA destabilization through deadenylation, or even the removal of the protective 5′ cap [17]. Importantly, miRNAs play an active role in a series of (patho)physiological mechanisms, including the control of inflammatory responses, which can be associated with autoimmunity or not [12,18,19,20,21,22,23]. 

Over 100 different types of arthritis have been described and miRNAs are implicated in some of them, as the literature, thus far, indicates [24,25]. However, osteoarthritis (OA) has increasingly attracted research interest because it is the most common form of arthritis [26]. Furthermore, the role of miRNAs in experimental models of arthritis and autoimmune diseases, including rheumatoid arthritis (RA), has been reported [27,28,29] and reviewed elsewhere [30,31,32]. Hence, in this review, we sought to discuss the role of miRNAs in the pathogenesis of two common types of non-autoimmune inflammatory arthropathies, OA and gouty arthritis, based on commonalities and correlations between these two different pathological entities: Gouty arthritis is associated with disturbed metabolic pathways, i.e., hyperuricemia, due to unbalanced purine nucleotide metabolism or excretion of uric acid by the kidney [33]. On the other hand, OA has been characterized as the fifth component of metabolic syndrome (MetS) [34]. More importantly, a positive correlation among hyperuricemia and MetS in different ethnicities has been found [35,36,37]. miRNAs are key controllers of chondrogenesis and the pathogenetic process of OA development [38,39], and they are interrelated with different metabolic routes [40,41,42]. In light of these data, the following paragraphs illustrate the involvement of miRNAs in pathways associated with both pathogenetic aspects of OA and gouty arthritis (from joint damage to pain), as well as their possible future exploitation in therapeutics for these inflammatory arthropathies.

## 2. OA and Gouty Arthritis: Overview of Pathophysiological Features

OA is a degenerative arthropathy associated with being overweight [43], which affects multiple tissues of the joints, including the cartilage, the subchondral bone, as well as the synovial membrane [44,45,46]. Consequently, in what follows, we will illustrate the role of miRNAs in different cell types in order to provide a global view of the pathogenesis of OA. Cartilage erosion and osteophytes at the cartilage–bone interface (osteophytosis) are pathognomonic features of this disease [47,48]. Synovial membrane inflammation, endochondral ossification, and subchondral bone sclerosis are well-known radiographic findings of OA, while the existence of a self-sustained hypoxia-inducible factor 2 alpha/Zn^2+^/zinc transporter ZIP8/metal-regulatory transcription factor 1 (HIF-2a/Zn^2+^/ZIP8/MTF1) catabolic circuit is a key molecular characteristic in this arthropathy [49,50,51]. Decreased levels of a major component of cartilage, the proteoglycan aggrecan [52], is a prominent biochemical feature of OA.

At the cellular level, chondrocytes and subchondral bone osteoblasts (SBOs) undergo numerous physiological alterations in OA: The regenerative capacity of OA chondrocytes is lost and OA chondrocytes acquire a “senescence-associated secretory phenotype” (SASP) characterized by the secretion of interleukin-1 (IL-1) and interleukin-6 (IL-6), as well as matrix metalloproteinase-3 (MMP-3) and -13 (MMP-13), which is largely induced by reactive oxygen species (ROS) [53]. In addition, lipid homeostasis is deregulated in chrondrocytes [54]. On the other hand, OA SBOs display a mineralization defect and altered profile of gene expression, which can be restored through miRNA mimics (i.e., synthetic miRNA-590-5p, miRNA-211-5p, miRNA-199a-5p, and miRNA-199a-3p), as evidenced in a rat model for OA. In fact, comparison among sclerotic and non-sclerotic bone specimens from OA sufferers demonstrated the existence of a molecular “signature” of eight miRNAs which possibly target 732 different transcripts, according to Ingenuity Pathway Analysis (IPA) bioinformatic analysis [55].

For many years, the prevailing view has been that inflammation and immune system components do not largely contribute to the pathogenesis of OA, which was thought to result from “wear and tear” processes. Yet, this was proven to be completely wrong [56]. Not only are human OA chondrocytes positive for components in miRNA biosynthesis (Dicer-1 and argonaute 2–4 (AGO2-4)) [57], but it is widely accepted that OA pathogenesis not only involves alterations in structural components of the joints, but also inflammatory and miRNA-dependent routes affecting different cellular processes, including lipid metabolism and autophagy [34,58,59,60]. Of note, RNA sequencing analysis revealed that human OA chondrocytes express several miRNAs that might play a role in OA, including the recently-identified miRNA-3085 [61]. Interestingly, miRNAs are implicated in pathways associated with altered lipid metabolism [54], as well as in routes possibly explaining the molecular basis of diabetes-induced OA [62].

Importantly, in silico analysis revealed the existence of distinct miRNA expression profiles, distinguishing OA from RA; even in the case that a single molecule (e.g., the transcription factor Ets-1) is predicted to be commonly targeted by miRNAs that display differential expression in OA and RA, this commonly-regulated molecule is postulated to be implicated in the pathogenesis of these different arthropathies through the modulation of different proteins [63]. 

On the other hand, gouty arthritis is a crystal-induced arthritis, i.e., a disease associated with the sterile inflammation of articular structures due to the deposition of crystals of monosodium urate (MSU). It may involve a single or multiple joints, and it progresses over four phases, i.e., asymptomatic hyperuricaemia, acute gout, intercritical gout, and chronic tophaceous gout [64,65]. Gouty arthritis can be misdiagnosed as rheumatoid arthritis, putting the lives of patients, simultaneously suffering from gout and other severe diseases, in jeopardy if it is not treated [66]. The identification of novel diagnostic markers with discriminative potential is, therefore, eagerly awaited. 

Toll-like receptor 2 (TLR2) and 4 (TLR4) are critical players in the phagocytic ingestion of MSU crystals, while cluster of differentiation 14 (CD14), a TLR2/4-interacting adaptor molecule, which is engaged by MSU crystals, is crucial for the MSU-induced secretion of interleukin-1 beta (IL-1β) and their inflammagenicity, without impacting their uptake by macrophages. Production of IL-1β involves the proteolytic maturation of pro-IL-1β by caspase-1 upon activation of a macromolecular complex known as NACHT, LRR and PYD domains-containing protein 3 (NLRP3) inflammasome [67,68]. In turn, ROS formed by nicotinamide adenine dinucleotide phosphate (NADPH) oxidase and purinergic receptor P2X7-dependent signaling can positively regulate NLRP3 inflammasome [68]. The uptake of MSU crystals by residual macrophages in inflamed tissue stimulates a series of inflammatory pathways that lead to the local recruitment of leukocytes (chemotaxis), possibly through endothelial activation [68]. Although the involvement of TLRs, NLRP3 inflammasome, and IL-1β in the MSU-induced inflammatory reaction is well-known, there are no miRNAs discovered, thus far, directly targeting TLRs, IL-1β, chemokines, and NLRP3 in gout [69].

## 3. OA and Overweight: A Perplexed Interplay

High throughput analysis of OA cartilage samples, employing miRNA array analysis, revealed that the expression of distinct miRNAs, exemplified by miRNA-22 and miRNA-103, is deregulated in OA. In fact, a set of 16 miRNAs, undergoing either up- or down-regulation, characterizes this pathological condition. The physiological importance of the deregulated miRNA world came into the foreground when these miRNAs were found to correlate with other clinicopathological parameters, or to modulate target genes associated with different processes, including biomechanical transduction, bone morphogenesis, and lipid metabolism. Specifically, a positive correlation among the body mass index (BMI, a measure of obesity) and the up-regulated miRNAs, miRNA-22 and miRNA-103, was reported. On the contrary, an inverse correlation for those miRNAs of which expression is negatively modulated in OA (miRNA-25, miRNA-29a, and miRNA-337) and BMI was observed [70]. These findings pinpoint the pathogenetic link between obesity and articular cartilage erosion. Elevated expression of miRNA-483 was demonstrated to be another characteristic feature of OA, which was validated using Northern blotting [70].

The aforementioned apparently “simplistic” interconnection between overweight and OA can be further molecularly subdivided into (i) OA related to biomechanical signaling routes; (ii) OA related to obesity and inflammation, and, finally; (iii) OA related to obesity and deregulated lipid metabolism. In fact, miRNAs are known to be implicated in all of these molecular subdivisions related to excess weight. First, the expression patterns of several miRNAs (miRNA-221, miRNA-222, and miRNA-365) display mechanoresponsiveness [71,72], while a mechanoregulatory protein, integrin alpha-5, the levels of which are correlated with BMI, was identified as a target of miRNA-25 that is down-regulated in OA specimens [70]. Second, miRNAs and their targets, adipokines, are key mediators of “metaflammation”, a type of inflammation triggered by imbalanced cellular energetics [73]. Third, proteomic profiling, conducted by Iliopoulos and colleagues [70], identified peroxisome proliferator-activated receptor alpha (PPAR-alpha), a miRNA-22 target, as a protein that is negatively regulated in OA. Importantly, it was found that the expression of PPAR-alpha, and those of IL-1β (a cytokine that seems to be repressed by PPAR-alpha in chondrocytes), also correlate with BMI [70]. These data, along with the fact that PPAR-alpha functions as a crucial regulator of lipid metabolism and obesity [74], suggest that miRNAs control all physiological aspects through which overweight pathogenetically contributes to OA, i.e., the biomechanics associated with joint overloading, as well as inflammatory pathways and deregulated lipid metabolism.

Interestingly, a negative and positive correlation of the BMI of OA patients with miR-26a and nuclear factor kappa B (NF-kB), respectively, has been reported. In primary chondrocytes derived from mice fed with a high fat diet, levels of miR-26a were decreased in comparison to mice fed with a standard chow diet, whereas, in contrast, the levels of the transcripts of pro-inflammatory cytokines were increased. In obesity-related chondrocytes, miR-26a counteracts the positive regulation of NF-kB by saturated non-esterified fatty acid (NEFA), while NF-κB functions as a suppressor of miR-26a [75].

The levels of miRNA-27a are found to be decreased in OA chondrocytes in comparison to normal chondrocytes [76]. Intriguingly, decreased expression of miRNA-27a has been reported to be a molecular feature of mature adipocytes in obesity [77]. Therefore, one might presume that deregulated miRNA-27a-dependent signaling accounts for a mechanistic link between OA and overweight. Additional studies are needed to address whether this hypothesis holds true, and to investigate a possible connection between miRNA-27a and miRNA-33a, which, as outlined above, is related to the altered metabolic pathways observed in OA [34]. In addition, it would be interesting to investigate whether miRNA-driven alterations in chondrocyte lipid metabolism are associated with changes in adipose tissue homeostasis, which could collectively contribute to the osteoarthritic phenotype.

## 4. miRNA-Associated Pathways in OA

### 4.1. Metabolic Pathways

In OA, chondrocytes are characterized by the decreased expression of proteins taking part in reverse cholesterol transport (RCT), such as apolipoprotein A1 (ApoA1), and by lipid accumulation [54,78]. Transcription factor sterol regulatory element binding protein 2 (SREBP-2) and miRNA-33a are fundamental in the orchestration of sterol metabolism [79]. A SREBP-2 variant, namely SREBP-2 G/C, has been found to occur at high frequencies in Greek patients with OA. In normal chondrocytes, this variant dampens the transforming growth factor beta (TGF-β)-induced expression of aggrecan, thereby highlighting the importance of metabolic circuits in OA pathogenesis [52]. 

miRNA-33a, a key modulator of lipid metabolism [80], is a central player, linking OA pathogenesis with deregulated metabolism. As a matter of fact, miRNA-33a is produced by a nested gene, lying within intron 16 of human SREBP2, and both of these molecules are up-regulated in OA chondrocytes. miRNA-33a and SREBP-2 are subjected to common positive transcriptional regulation by TGF-β1, while miRNA-33a is essential for the TGF-β1-dependent expression of SREBP-2 itself. When normal chondrocytes are transfected with a miRNA-33a mimic, MMP-13 expression is increased, whereas, in contrast, levels of ApoA1 drop, although ApoA1 is not a direct miRNA-33a target. ATP binding cassette subfamily A member 1 (ABCA1), which is implicated in cholesterol efflux, is also decreased by miRNA-33a [34]. Given the importance of ApoA1 in RCT [81], and the role of MMP-13 in the acquisition of the osteoarthritic phenotype by chondrocytes [82], miRNA-33a emerges as a central player linking OA pathogenesis to deregulated metabolism [34].

Taking another step towards linking miRNAs with the modulation of metabolic networks and degenerative skeletal disorders, these post-transcriptional regulators have been postulated to be somehow involved in the progression of diabetic OA; however, rigorous evidence supporting this notion is lacking [62].

### 4.2. Inflammatory Networks, Degenerative Enzymes, and Zinc as the Molecular Link among Them

#### 4.2.1. Inflammatory Signaling Pathways, MMPs, and A Disintegrin and Metalloproteinase with Thrombospondin Motifs Enzymes

Comparison of the miRNA expression profiles among cartilage, as well as bone obtained from OA and non-OA individuals, indicated that there are certain miRNAs that undergo a striking OA-specific up- or down-regulation. Specifically, in both cartilage and bone specimens from OA patients, miRNA-9 and miRNA-98 were detected at 8- and 23-fold higher levels compared to control samples, while miRNA-146 was found to be 14 times more abundant in OA samples than in control specimens. Bioinformatics predicted that all of the miRNAs mentioned above affect gene targets, which are implicated in inflammatory signaling pathways, such as the complement system, the NF-kB pathway, and leukocyte extravasation [83]. In a more recent study employing a surgical model of rat OA, it was found that miRNA-210 levels drop in diseased animals in comparison to sham-operated animals. When miRNA-210 is ectopically expressed via the administration of lentiviral constructs in the joints of OA rats, both the secretion of pro-inflammatory mediators and activation of the NF-kB pathway are prevented by miRNA-210 [84]. Similarly, miRNA-502-5p has also been found, through in vitro experimentation, to counteract NF-kB signaling. Mechanistically, this involves miRNA-502-5p-mediated inhibitory targeting of tumor necrosis factor receptor-associated factor 2 (TRAF2) [85]. The miRNA-29 family, of which expression is regulated throughout the development/progression of OA, also dampens NF-kB-mediated signalling [86].

An inflammatory circuit which seems to be etiopathologically linked to OA, is a miRNA-149-dependent one, where a drop in miRNA-149 levels in chondrocytes is correlated with an elevation in the expression of a set of cytokines (IL-6, IL-1β, and tumor necrosis factor alpha (TNF-α) [87]. A microarray-based analysis of miRNAs in human OA chondrocytes, stimulated by IL-1β, showed that the expression of 36 miRNAs were altered, with the majority of them (35 out of 36) being downregulated and affecting inflammatory networks [88].

Several studies have demonstrated that there are certain miRNAs that influence the expression of degenerative enzymes, such as MMP-13 and a disintegrin and metalloproteinase with thrombospondin motifs (ADAMTS) extracellular matrix proteases. A set of ADAMTS enzymes (ADAMTS4, ADAMTS5, ADAMTS7, as well as ADAMTS12) are controlled by miRNA-105, plausibly via targeting Runt-related transcription factor 2 (Runx2). In turn, miRNA-105 is negatively modulated by the p65 subunit of NF-kB upon stimulation of chondrocytes with fibroblast growth factor 2 (FGF2), suggesting that an FGF2/p65/miRNA-105/Runx2/ADAMTS pathway is critically implicated in the etiopathogenesis of OA. Significantly, it has been reported that there is an inverse correlation among miRNA-105 and Runx2, as well as ADAMTS [89]. Catabolic effects on the cartilage matrix, through the regulation of ADAMTS and MMPs, as well as regulation of Mothers against decapentaplegic homolog 3 (Smad3) synthesis, are also possibly exerted by miRNA-16-5p, which is found to be upregulated in cartilage specimens from OA sufferers compared to control samples [90].

A large-scale analysis demonstrated that miRNA-27b, as well as 41 other miRNAs undergo down-regulation in human OA chondrocytes in response to IL-1β, a key cytokine in OA development. IL-1β-dependent regulation of miRNA-27b is of great pathophysiological importance, as in silico analysis and luciferase reporter assays indicated that MMP-13 is targeted by this miRNA, with NF-kB functioning as a negative and positive modulator of miRNA-27b synthesis and MMP-13 secretion, respectively [57]. miRNA221-3p is another miRNA that can be suppressed by IL-1β. Interestingly, miRNA221-3p has recently been reported to be downregulated in synovial fibroblasts derived from subjects suffering from OA of the temporomandibular joint (TMJOA). This results in the derepression of the expression of its target, transcription factor Ets-1, and the consequent stimulation of the synthesis of the degradative enzymes, matrix metalloproteinase-1 (MMP-1) and 9 (MMP-9) [91]. miRNA-222, the expression of which is decreased in OA chondrocytes, is known to downregulate MMP-13 via targeting of histone deacetylase 4 (HDAC-4), and in mice where the osteoarthritic phenotype is surgically induced through destabilization of the medial meniscus (DMM), the overexpression of miRNA-222 in the knee in these animal models restrains cartilage damage [92].

The findings of another study imply that miRNA-602 and miRNA-608 could be exploited therapeutically in OA to suppress MMP-13. In fact, experimentation in animal models, as well as in different cell types (including chondrocytes stimulated with IL-1β), showed that the expression of MMP-13 is actually positively regulated by sonic hedgehog (SHH), the expression of which, in turn, is repressed by miRNA-602 and miRNA-608 [93]. ADAMTS5 (aggrecanase) is a miRNA-140 target, which finds application in modelling OA in animals. Silencing the expression of miRNA-140 in mice results in the establishment of an age-related mouse model of OA, where the aggrecan content in the cartilage matrix is decreased by the age of 12 months in miR-140−/− mice due to the derepression of the expression of ADAMTS5. On the other hand, miR-140-overexpressing transgenes are endowed with resistance to the development of arthropathy [94]. Part of the bone-protecting role of miRNA-140 is also possibly attributed to its ability to suppress the expression of MMP-13 in chondrocytes in a negative-feedback regulatory circuit [95].

miRNA-139 is an IL-1β-inducible miRNA that displays a chondral lesion-specific pattern of expression in OA, which seems to stimulate cartilage matrix catabolic processes via positively regulating the expression of the enzymes MMP-13 and ADAMTS4. Additionally, miRNA-139 exerts pro-apoptotic effects in human OA chondrocytes [96]. Employing the mono-iodoacetate-induced arthritis (MIA) rat model for OA, it was demonstrated that miRNA-101 stimulates the expression of a series of genes associated with extracellular matrix (ECM) degradation, including ADAMTS1 and ADAMTS5, as well as the expression of several cytokines. The usage of this animal model, which faithfully recapitulates human OA, showed that the intra-articular delivery of miRNA-101 mimic or miRNA-101 inhibitors by means of adenoviral vectors is a promising strategy to modulate cartilage erosion [97].

More recently, it was reported that miRNA-142-3p impedes the progression of OA in animals by acting as a negative modulator of the high mobility group box 1 protein (HMGB1)/NF-kB pathway [98]. Consistent with the anti-arthritic and chondroprotective properties of the bioactive compounds of green tea, exemplified by epigallocatechin-3-*O*-gallate (EGCG) [99,100], this green tea polyphenol engages a miR-199a-3p-dependent pathway to repress the expression of cyclooxygenase-2 (COX-2) and the synthesis of prostaglandin E_2_ (PGE_2_) in chondrocytes treated with IL-1β [101].

Interestingly, an inverse correlation among the expression pattern of miRNA-146α and MMP-13 is observed when the levels of these two molecules are evaluated in grade I and grade II OA, while miRNA-146α expression drops when OA progresses to more advanced stages, according to the Mankin scoring system. In vitro, it was demonstrated that miRNA-146α is increased in response to IL-1β. A role of miRNA-146α in cartilage homeostatic circuits (modulation of anabolic versus catabolic signals) or the induction of early OA by miRNA-146α have therefore been proposed as possible scenarios [102]. In line with these findings, in human chondrocytes, miRNA-146α suppresses the expression of MMP-13, as well as those of ADAMTS5 [103]. On the contrary, a positive correlation among miRNA-483 and MMP-13 in an experimental mouse model of OA has been found, implying the role of miRNA-483 in OA pathogenesis [104].

#### 4.2.2. Zinc at the Crossroads of Inflammation and Cartilage Erosion

miRNA-488 is a TGF-β3-inducible miRNA, of which expression is decreased in OA chondrocytes compared to normal chondrocytes, and that targets ZIP8 (SLC39A8), a Zn^2+^ importer. Significantly, the suppression of ZIP8 expression contributes to the preservation of cartilage integrity in experimental models of OA [105]. This is consistent with the link between zinc homeostasis and OA. In fact, a zinc/ZIP8/MTF1 pathway that functions as a “molecular bridge” among inflammation and cartilage erosion has been described, as ZIP8 is an IL1β-inducible transporter, while the Zn^2+^-responsive transcription factor MTF1 positively regulates the expression of MMP-3, MMP-13, and ADATMTS5 [50,106,107]. Hence, the miRNA-488/ZIP8/MTF1/MMPs-ADAMTS5 axis is critical for the pathogenesis of OA.

### 4.3. TGF-β/Smad, Wingless , and Vascular Endothelial Growth Factor Pathway

A microarray-based, large-scale analysis identified the signatures of six miRNA, of which levels are increased in normal chondrocytes of human origin relative to OA chondrocytes (miRNA-1227, miRNA-576-5p, miRNA-149*, miRNA-634, miRNA-641, and miRNA-582-3p) and a single miRNA, namely miRNA-483-5p, which is selectively up-regulated in OA chondrocytes [108]. The finding of increased miRNA-483-5p in OA chondrocytes is in line with the results of Iliopoulos et al., who reported that miRNA-483 appeared to be up-regulated in diseased cartilage derived from OA patients [70]. The signalling circuits that these miRNA:target pairs participate in include Wingless (Wnt) and TGF-β/Smad pathways, which are pathogenetically related to OA, or are essential for the differentiation of chrondrocytes and in the onset/progression of OA [108]. Intriguingly, a negative correlation among miRNA-483 and the expression of TGF-β, as well as those of the TGF-β superfamily member bone morphogenic protein 7 (BMP7) in murine experimental OA, has been reported. The levels of both miRNA-483 and miRNA-483*, which reside within an intronic region of the insulin-like growth factor 2 (IGF2) locus, undergo an increase upon surgical intervention in an animal model of OA, and, hence, they are thought to be pathogenetically implicated in OA, irrespective of IGF2 [104]. A more recent study demonstrated that many of the Wnt pathway components (e.g., dishevelled segment polarity protein 3 (DVL3) and frizzled class receptor 5 (FZD5)) are directly targeted by miRNA-29 family members, of which the regulation of expression in cartilage seems to be important during the course of OA development. In fact, the miRNA-29 family is suppressed by transcription factor SRY-box 9 (SOX9), whereas, in turn, the miRNA-29 family dampens Wnt- and Smad-mediated signalling [86]. In human chondrocytes, Smad3 has been identified as a target of miRNA-16-5p, which has been incriminated in the development of OA [90]. According to a more recent study, Smad3 is also directly targeted by miRNA-23a-3p, of which expression in OA cartilage is elevated due to CpG island promoter hypomethylation. The net result of this miRNA-23a-3p/Smad3-dependent pathway is the decreased type II collagen/aggrecan content of the cartilage extracellular matrix in OA [109].

Another study demonstrated that in OA a prominent feature is the altered expression of two miRNAs, miRNA-140-5p and miRNA-455-3p [110]. A reciprocal regulation among miRNA-455-3p and TGF-β pathways—TGF-β influences miRNA-455-3p levels, while miRNA-455-3p dampens Smad2/3-mediated signaling, thereby possibly contributing to cartilage erosion—has been reported. Smad2, Chordin-Like 1 (CHRDL1), and Activin Receptor Type IIB (ACVR2B) have been identified as direct miRNA-455-3p targets, which are related with the TGF-β pathway [110]. On the other hand, miRNA-140 displays an almost chondrocyte-specific pattern of expression and controls pathways that are essential for unperturbed bone morphogenetic factor (BMP)/Smad signaling in an evolutionarily conserved manner, from mouse to zebrafish [111]. Intriguingly, both miRNA-455-3p and miRNA-140-5p are derived from intronic miRNA precursors that reside within collagen type XXVII alpha 1 (COL27A1) and WW domain containing E3 ubiquitin protein ligase 2 (WWP2) locus), respectively [110,111]. Of note, the spatiotemporal pattern of COL27 expression is known to play a crucial role in human skeletogenesis [112], while WWP2 is critical for craniofacial development [113]. In other words, the coding regions of miRNAs that are thought to be implicated in OA pathogenesis reside within host introns of genes that are critical for skeletal development.

In a study employing IL-1β-stimulated primary rat chondrocytes and a surgical rat model of OA where OA develops due to joint destabilization, miRNA-146α was found to be elevated in OA rats in comparison to sham-operated animals [114]. Smad4 transcription factor was characterized as a direct target of miRNA-146α, and its targeting by miRNA-146α was found to be essential for the ability of miRNA-146α to upregulate the expression of vascular endothelial growth factor (VEGF) in vitro and in vivo. In addition, miRNA-146α was found to suppress TGF-β signaling and to trigger chondrocyte apoptosis. Hence, it is postulated that miRNA-146α contributes to the OA phenotype by controlling TGF-β and VEGF via inhibiting their common mediator, Smad4. miRNA-146α-induced increase in VEGF in OA cartilage could contribute to abnormal angiogenesis and endochondral ossification during OA progression [114].

### 4.4. Autophagy

As evidenced by experiments in different mouse models of OA, autophagy is a homeostatic mechanism in cartilage, where it displays a constitutive pattern of expression, but is deregulated in favor of apoptotic cell death in OA [115]. Interestingly, a recent study identified miRNA-155 (a miRNA upregulated in OA) as a key negative modulator of the autophagic flux in human chondrocytes. miRNA-155 suppresses a series of autophagy-associated molecules exemplified by Atg proteins participating in the formation of the autophagosome, forkhead box O3 (FoxO3), as well as the autophagy inducer Unc-51-like kinase 1 (Ulk1)). Hence, miRNA-155 has been linked to the development of OA [116]. Since aging increases the risk for OA development, the decrease in the autophagic flux observed in OA chondrocytes is an aging-associated event [53].

### 4.5. Nociception

In human joint synoviocytes, miRNA-146α controls not only the expression of both cartilage matrix-degrading enzymes, but also those of inflammatory mediators associated with nociception. More importantly, it was reported for the first time that, in comparison to non-OA rats, OA animals experiencing pain express lower levels of miRNA-146α in their dorsal root ganglion (DRG) and spinal dorsal horn [103]. In addition, the role of miRNA-146α, not only in the pathogenesis of OA, but also in its clinical manifestations (i.e., chronic pain) was substantiated by the fact that miRNA-146α dampens the expression of a set of inflammatory molecules associated with pain perception in human glial cells, as well as the expression of transient receptor potential cation channel subfamily V member 1 (TRPV-1) [103]; an ion channel, which has attracted the interest of many pharmaceutical companies as a putative target of analgesic agents [117].

### 4.6. Mesenchymal Stem Cell-Associated Pathways

**Figure 1:**
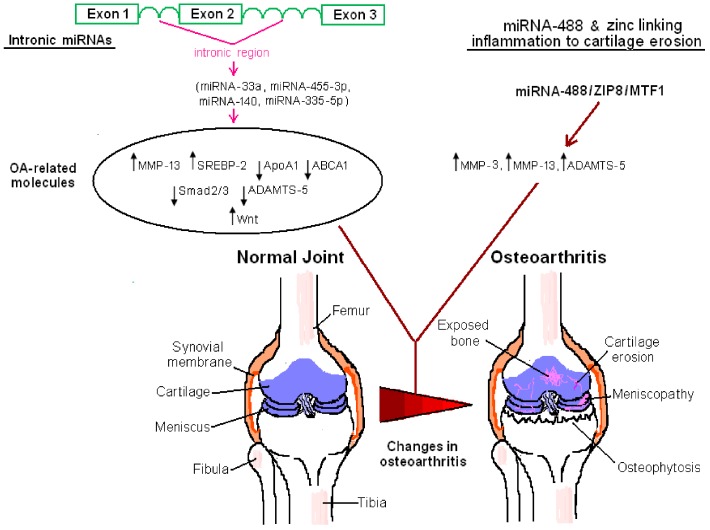
Intronic microRNAs (miRNAs) and the miRNA-488/ZIP8/MTF1 pathway are critically involved in the pathogenesis of osteoarthritis (OA). Distinct miRNAs (miRNA-33a, miRNA-455-3p, miRNA-140, and miRNA-335-5p) reside within intronic regions of human genes and they modulate the expression of a series of molecules either positively (upward pointing arrows) or negatively (downward pointing arrows) associated with the development of osteoarthritis. These molecules include proteases responsible for the degradation of cartilage matrix (matrix metalloproteases (MMPs), A Disintegrin and Metalloproteinase with Thrombospondin Motifs 5 (ADAMTS5)), molecules related with cholesterol synthesis and efflux in OA chondrocytes (sterol regulatory element binding protein 2 (SREBP-2), apolipoprotein A1 (ApoA1), and ATP binding cassette subfamily A member 1 (ABCA1)), or they affect Mothers against decapentaplegic homolog 2/3 (Smad2/3)- and Wingless (Wnt)-dependent signalling. On the other hand, miRNA-488 influences a zinc transporter ZIP8/ metal-regulatory transcription factor 1 (ZIP8/MTF1)-mediated route, which eventually upregulates a set of molecules that are implicated in the enzymatic erosion of articular cartilage, i.e., MMP-3, MMP-13, and ADAMTS5. These molecular events largely contribute to the OA phenotype through promoting pathological changes; erosion of cartilage and meniscus, denudation of the subchondral bone and osteophytosis (see the text for details). Other miRNA-dependent, as well as miRNA-independent, pathways are involved in the pathogenesis of OA. However, they are not depicted here for reasons of simplicity. Intronic miRNAs reside in introns from different loci. A common graph of three exons has been drawn here for reasons of clarity. Key anatomic domains of the knee and their alterations during OA development are indicatively depicted. These molecular events are not only associated with knee osteoarthritis but they may also be localized in other joints.

A large-scale study investigating the differential pattern of miRNAs expressed in mesenchymal stem cells (MSCs) derived from the bone marrow of OA and healthy donors provides insight into the possible role of MSCs in OA. A distinguishing feature of OA MSCs is the down-regulation of miRNA-335-5p. This is of major functional importance, since this miRNA targets the Wnt pathway antagonist Dickkopf Wnt signaling pathway inhibitor 1 (DKK1), thereby stimulating Wnt; a pathway that is interrelated with osteogenesis [118]. Given the role of unbalanced subchondral bone remodeling in the pathogenesis of OA, and the putative therapeutic value of manipulating MSCs in this process [119], miRNA-335-5p-dependent pathways could possibly be therapeutically harnessed in regenerative medicine. Of note, the miRNA-335-5p-coding region is actually an intronic miRNA (Figure 1). It is harbored within an intron by a gene, mesoderm specific transcript (MEST) [118]. On the other hand, miRNA-30a directly targets the Notch ligand Delta-like 4 (DLL4), thereby stimulating MSCs to express indicators of chondrogenic differentiation [120]. The usage of miRNAs for the stimulation of MSCs, to engage in a differentiation program, is a concept that is gaining ground in regenerative medicine and OA therapeutics [121].

## 5. miRNA-Associated Pathways in Gouty Arthritis

Analysis in mononuclear cells isolated from individuals suffering from gouty arthritis showed that miRNA-155 is expressed at higher levels in synovial fluid mononuclear cells (SFMCs) of patients in comparison to peripheral blood mononuclear cells (PBMCs) derived from healthy subjects. With the aim of unveiling a possible etiological link between miRNA-155 and gouty arthritis, it was found that the exposure of PBMCs from healthy participants to MSU crystals resulted in the induction of the expression of this miRNA. Src homology 2 (SH2) domain-containing inositol-5-phosphatase 1 (SHIP-1) levels were found to be lower, both in SFMCs of patients and in PBMCs of healthy controls on treatment with MSU crystals than in MSU-untreated PBMCs of healthy individuals. This is consistent with the fact that the immunoregulatory protein SHIP-1 is directly targeted by miRNA-155. SHIP-1 displays anti-inflammatory actions and the miRNA-155/SHIP-1 route is thought to fuel inflammation in gouty arthritis [122]. 

It is noteworthy that in THP-1 human monocytic cells, the decrease in SHIP-1 levels was found to coincide with a decrease of a molecule that is critically involved in inflammation—NF-kB inhibitor (IkB)—upon challenge with MSU crystals. miRNA-155-transfected or MSU-challenged THP-1 cells display a profound alteration in their pro-inflammatory secretory activity, exemplified by the elevation of IL-1β and TNF-α extracellular release, as evidenced by a multiplex immunoassay, termed Luminex. In line with the in vitro data, miRNA-155 is increased in the murine gout model. Importantly, in gouty arthritis clinical synovial specimens, the expression of SHIP-1 was reported to be scarce, which is in striking contrast to OA samples, which were positive for SHIP-1 [122]. The aforementioned data favor the notion that miRNA-155-dependent signaling positively regulates inflammation in gouty arthritis. In addition, these data pave the road for the identification of novel discriminative biomarkers for diagnosing gouty arthritis and other inflammatory arthropathies. A more recent study suggests that miRNA-146α halts inflammation evoked by MSU crystals; in fact, it suppresses the expression of a series of inflammatory mediators (monocyte chemoattractant protein-1 (MCP-1), TNF-α, interleukin 8 (IL-8), IL-1β). However, miRNA-146α is somehow lost throughout the acute response to these pro-inflammatory irritant particles [123].

## 6. miRNA-Dependent Pathways as Therapeutic Targets in OA and Gouty Arthritis: At the Juncture of Biotechnology and Traditional Medicine

Agents used for the management of pain and inflammation, or as preoperative regimens in OA patients, may cause undesired side effects [124,125,126]. Hopefully, miRNA-based strategies hold promise for OA therapeutics [127], and could be used as an alternative, and perhaps safer, treatment option. miRNAs are actually nucleic acids that can be targeted using oligos that exhibit complementarity with the corresponding targets, to a lesser or greater extent in a similar way to antisense oligonucleotides (ASOs), which are used to silence mRNA targets. This stategy is termed miRNA antagonism, and complementary oligos are known as “antagomiRs”. Silencing miRNAs typically leads to the derepression of genes that are negatively modulated by miRNAs. The liver-specific miRNA-122 was actually among the first miRNAs to be targeted for silencing. In vitro, miRNA-122 was first targeted by an 2′-*O*-methylated ribonucleotide [128], while in vivo, miRNA-122 has been effectively and long-lastingly silenced by Krützfeldt and colleagues through enrolling a phosphorothioate (PS)-modified, cholesterol-conjugated 2′-*O*-methylated RNA molecule [129]. In the latter case, and from a historical perspective, the aforementioned researchers named the cholesterol conjugates of phosphorothioated, 2′-*O*-methylated RNA as “antagomiRs”. Since 2005, various chemically-modified complementary oligos have been designed to knock down the expression of miRNAs. Consequently, the term “antimiRs”, which includes antagomiRs, can be used to describe all the chemically divergent molecules targeting miRNAs. 

The pharmacokinetic properties (e.g., binding affinity and resistance to nucleases) of antimiRs can be optimized via a series of modifications, such as ribose sugar modification at the 2′ position, the introduction of PS linkages into the phosphodiester backbone, the replacement of the sugar moieties with morpholino rings, or even the introduction of a 2′-*O*,4′-*C*-methylene bridge to generate bicyclic ribonucleotides, termed “locked nucleic acid” (LNA) molecules. The latter RNA analogues are characterized by the formation of particularly stable duplexes with their targets, exhibiting high melting temperatures (Tm values) [130]. Of note, miravirsen (SPC3649), an agent inhibiting hepatitis C virus (HCV), was the first anti-miRNA drug to enter clinical trials. It is a nuclease-resistant phosphorothioate-modified LNA oligo, which is complementary to the 5′ end of miRNA-122; a liver-specific miRNA, which is essential for HCV virulence [131]. A phase IIa clinical trial shows that miravirsen displays satisfactory, long-term beneficial effects in chronically genotype I, HCV-infected subjects in a dose-dependent manner, without any signs of development of viral resistance [132]. Interestingly, small-length LNAs, the so-called “tiny LNAs”, are complementary to the seed sequence of miRNAs, and can achieve the selective, long-term inhibition of a whole family of miRNAs sharing common seeds [133].

Moreover, antimiRs can be delivered either as naked species or as nanoparticles. The usage of nanotechnology may largely affect the biodistribution of an antimiR agent [134]. Another strategy to sequester miRNAs is the usage of the so-called “miRNA sponges”, introduced to biotechnology in 2007. miRNA sponges exhibit multiple (4–10), tandem binding sites for the miRNA target, separated by spacers. A whole miRNA family can be targeted by miRNA sponge constructs, since they block the functions of miRNAs with a common seed sequence. miRNA sponges can be delivered to cells via viral vectors, such as lentiviruses. For this reason, miRNA sponges can achieve long-term expression of the miRNA construct, as has been shown in different animal species. In contrast, antagomiRs have to be repeatedly administered at high doses in order to achieve long-term miRNA inhibition. An additional advantage of miRNA sponge technology is that, unlike cholesterol-conjugated antagomiR oligos, which are accumulated in the hepatic parenchyma, the design of miRNA sponges may allow their expression in any tissue of interest [135,136].

Another miRNA-based treatment strategy involves the employment of the so-called “miRNA-MASKs”. These are single-stranded oligos carrying 2′-*O*-methyl modifications, which are designed to be complementary to the 3′ UTR of the target transcript, thereby occupying it and hindering its interaction with the corresponding miRNA. The net result is the blockage of miRNA function and the derepression of the expression of a gene of interest [137]. Another approach is the usage of “miRNA mimics”. These synthetic molecules are double-stranded oligos, which in contrast to miRNA-MASKs, mimic the sequence of a mature miRNA molecule, or even the sequence of pre-miRNA [138]. Alternatively, a pri-miRNA mimic can be designed to mimic the primary miRNA molecule. The latter constructs engage the endogenous miRNA biogenetic pathway upon their delivery, and they are effective in gene silencing [139]. Noticeably, miRNA mimics have been used to uncover the role of the miRNA-30b/ERG pathway in OA [140], as well as the regulatory function of miRNA-634 in chondrocyte metabolism [141].

Pre-clinical experimentation in animals suggests that some miRNA species are possible targets for therapeutic intervention in OA. For instance, lentiviral delivery of miRNA-222 into the knee of a surgical DMM mouse model of OA yielded encouraging results [92]. On the contrary, miRNA-21, which suppresses the TGF-β superfamily member growth/differentiation factor 5 (GDF-5), has been incriminated in the development of OA due to its anti-chondrogenic effects [142]. This finding is of major importance, since miRNA-21 has already been successfully employed in an in vitro assay evaluating the RISC loading inhibitory potency of different compounds [143]. Hence, the notion that miRNA-21 may serve as a therapeutic target in OA is being reinforced. Other miRNA species critically implicated in OA pathogenesis, such as miRNA-155, do not seem to be “targetable”: miRNA-155 integrity is required for the unperturbed primary and memory immune responses, as evidenced in mice [144]. Conceivably, the putative targeting of a specific miRNA for therapeutic purposes is not always an easy matter and raises various concerns.

Recently described biotechnological techniques, such as the clustered regularly interspaced short palindromic repeats (CRISPR), can advance the characterization of miRNAs, which specifically operate in chondrocytes and OA pathogenesis, as well as the perplexed signalling networks in which they participate. Further, enrolling robust methodologies for the validation of direct miRNA targets, such as the high-throughput sequencing of RNA isolated by cross-linking immunoprecipitation (HITS-CLIP), will hopefully contribute to the minimization of hybridization-dependent phenomena, which are major hurdles in clinical applications [145]. These methodologies in combination with certain drug delivery modes, such as the use of synthetic materials, can enable the development of novel and efficacious therapeutic approaches in OA and/or gouty arthritis and the improvement of different parameters that are crucial for miRNA-based therapeutics. These parameters include hybridization-independent issues, e.g., the phagocytic uptake of circulating miRNA-containing particles, or even the off-target effects observed when miRNAs carry chemical modifications [146].

**Figure 2:**
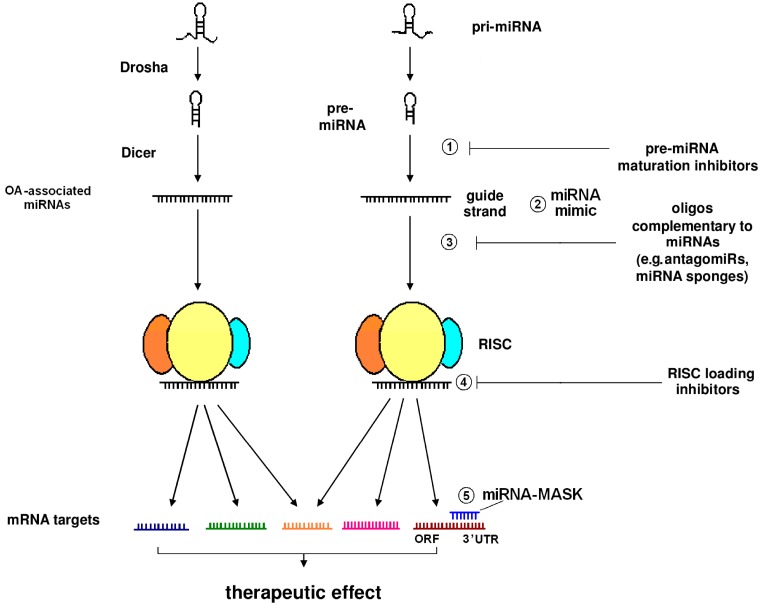
OA-associated miRNA-dependent pathways may be therapeutically targeted at multiple levels. Dicer-mediated processing of certain pre-miRNAs of the corresponding mature miRNA (guide strand) can be blocked via small molecules that serve as maturation inhibitors (level 1). miRNA mimics can be used to mimic the function of endogenous miRNA molecules (level 2). Oligonucleotides (oligos) complementary to miRNAs, such as antagomiRs and miRNA sponges, can interfere with miRNA:mRNA interactions through hybridization with target miRNAs (level 3). Chemically divergent small molecules, which block the assembly of RNA-induced silencing complex (RISC), designated as RISC loading inhibitors, have been also identified. These inhibitors do not affect argonaute 2 (Ago2) activity or pre-formed miRNA:Ago2 complexes (level 4). On the other hand, miRNA-masking (miRNA MASK) technology (level 5) involves the usage of single-stranded chemically modified oligos, which are complementary to the 3′ untranslated region (UTR) of a target transcript. Therefore, the interaction among miRNA and its target is prevented, and the expression of a gene of interest is derepressed. Overall, the net result of all of the above-mentioned putative therapeutic strategies is the modulation of the expression of different mRNAs that are associated with OA pathogenesis. Note that a miRNA may regulate multiple miRNA targets, while a distinct mRNA may be controlled by more than one miRNA. This phenomenon contributes to the generation of a complicated network of interplaying pathways. ORF: open reading frame; pri-miRNA: primary miRNA.

Interestingly, experiments have been carried out in order to study the molecular pathways underlying the therapeutic effects of herbal decoctions commonly used by Chinese medicine practitioners, such as the Xie-Zhuo-Chu-Bi-Fang (XZCBF) and the Xiezhuo Chubi Decoction (XZCBD). Data demonstrate that there are distinct miRNAs associated with herb-based therapeutics of hyperuricaemia, at least in a pre-clinical setting (hyperuricemic models). Certain miRNAs affect the expression of molecules critically implicated in uric acid handling, i.e., urate transporter 1 (URAT1)and xanthine oxidase (XO). In fact, traditional Chinese herb-based treatment with XZCBD has been reported to exert a suppressive effect on the expression of URAT1. This is possibly attributed to the XZCBD-induced upregulation of miRNA-34a, although more than 30 other miRNAs are also modulated by this formula at high doses [147]. According to a more recent study, miRNA-34a has been shown to counteract URAT1-mediated renal reabsorption of uric acid, thereby mitigating the disease phenotype in animals treated with XZCBF [148]. Furthermore, evidence has been provided that miRNA-448 modulates XO levels in an allopurinol-dependent manner [149]. In substantiation of the plausible importance of miRNAs in circuits associated with gout pathophysiology, URAT1 is crucial for the regulation of serum levels of uric acid [150] and XO is currently the mainstay pharmacological target in gout [151]. Overall, experimental evidence suggests that miRNA-dependent pathways are targeted by herbal formulations used in traditional medicine. In addition, miRNAs are possible targets for therapeutic intervention in OA, where their functions can be modulated by virtue of biotechnology advances, as depicted in Figure 2.

## 7. Conclusions and Future Perspectives

Inflammation and cartilage erosion in OA are linked by a miRNA-488-regulated Zn^2+/^ZIP8/MTF1 pathway, which affects the expression of MMPs-ADAMTS5. The prevailing view is that OA is a “wear and tear” process, which, in an oversimplified manner, is solely associated with cartilage erosion due to the cartilage matrix degeneration by catabolic enzymes (MMPs, ADAMTS enzymes). By taking advantage of high throughput screening and RNA sequencing technologies, and via experimental models of OA (surgical instability models or genetically engineered animals), it has been demonstrated that miRNAs not only simply act as regulators of inflammatory processes in the affected joints (e.g., chemotaxis), but also as key metabolic regulators at the juncture of the modulation of metabolism and inflammation (metaflammation), as well as in the control of metabolic pathways, which mechanistically link obesity to OA. Downregulated RCT, critically modulated by miRNA-33a, and deregulated autophagic flux, modulated by miRNA-155, are prominent molecular features of OA. From a structural genomics point of view, several miRNAs reside within the intronic regions of the human genome [152,153]. Certain OA-related miRNAs, i.e., miRNA-33a, miRNA-455-3p, miRNA-140, and miRNA-335-5p, are actually embedded within the intronic regions of human SREBP-2, COL27A1, WWP2, and MEST, respectively. The intronic class of miRNAs possibly displays biogenetic differences with regard to the exonic class of miRNAs in mammals [152]. Whether this intronic class of miRNAs differentially influences OA pathogenesis, compared to the exonic class of miRNAs, is a very interesting topic for future research. Another hallmark of OA is miRNA-335-5p downregulation in OA MSCs; something that can be therapeutically harnessed in the future, in the field of regenerative medicine.

In addition to OA, miRNAs are also central players in pathways associated with MSU-induced inflammation and gouty arthritis. The identification of miRNA(s) possibly targeting TLRs, IL-1β, chemokines, and NLRP3 in gouty arthritis is another major goal for the future. The etiopathological role of miRNA-155 is a commonality between OA and gouty arthritis, although, in OA, miRNA-155 functions as a suppressor of autophagy, whereas a miRNA-155/SHIP-1 pathway has been incriminated in gouty arthritis. Thus far, no such metabolism-associated miRNA has been discovered that links OA and gouty arthritis, although there is a positive correlation among hyperuricemia and MetS, and OA is being conceptualized as the fifth component of MetS. Thus, it would be interesting for researchers to address whether there is/are any common metabolism-related miRNA(s) playing a role in the pathogenesis of OA and gouty arthritis. This is a challenging task for the future.

Another future challenge for OA therapeutics would be to address whether there are OA-related miRNAs displaying evolutionary conservation of seed sequences, so that they could possibly be targeted by “tiny LNAs”. Moreover, interfering with miRNA-146α-dependent pathways controlling nociception in OA [103] might serve as a strategy to relieve pain. In fact, the idea that miRNA-mediated axes could be targeted for the management of pain has been gaining ground [154].

The studies mentioned above unequivocally indicate that miRNAs are interrelated with the pathogenesis of OA, as well as gouty arthritis. Even in the case that miRNAs are not etiopathologically associated with OA, their expression is somehow influenced throughout the OA development process, either as single non-coding molecules or as part of a miRNA “signature”. miRNAs could be clinically used (e.g., in a microarray-based format) as a feasible procedure for the specific assessment of OA, which could be less expensive than magnetic resonance imaging (MRI). More experimentation is required in this direction [155]. Validating a certain miRNA or miRNA sets, as non-invasive biomarker(s) in patients suffering from OA or gouty arthritis, using an established statistical method, such as receiver operating characteristic (ROC) curve analysis as has been attempted in other diseases [156,157], would therefore be another future goal. For instance, miRNA let-7e seems to be a negative predictor for severe OA, at least in Caucasians [155], and its validation as a biomarker is of great interest. Valuable data regarding the usefulness of distinct miRNAs as biomarkers at the pre-clinical level in animal models have been already provided [158]. However, ROC analyses are nodal statistical tools in clinical studies. Clinical data, properly processed in order to be statistically refined, are much-awaited, in order to propel the introduction of possible miRNA-based therapies into clinical practice.

It is worth mentioning that miRNAs are part of the underlying mechanism by which herbal decoctions, used by traditional Chinese medicine, act in the management of OA. A significant “take-home message” is that miRNA-mediated control plays a key role in the cellular responses to medical practices applied all over the world, from the West to the East. In addition, miRNAs are involved in the modulation of the inflammatory pathways activated in different types of arthropathies, whether they are evoked by crystal deposits or not.
